# P-1435. Association between Social Determinants of Health and Modifiable Cardiovascular Risk Factors in People with HIV in the V-EXTRA-CVD Study

**DOI:** 10.1093/ofid/ofae631.1609

**Published:** 2025-01-29

**Authors:** Laura Hmiel, Sandra Woolson, Elizabeth Strawbridge, Lewis S Musoke, Brigid Wilson, Nadine M Harris, Hayden Bosworth, Puja Van Epps

**Affiliations:** MetroHealth Medical Center, Cleveland, Ohio; Durham VA Health Care System, Durham, North Carolina; Durham VA Health Care System, Durham, North Carolina; Veterans Affairs Northeast Ohio Healthcare System, Cleveland, OH; VA Northeast Ohio Healthcare System, Cleveland, Ohio; Atlanta VA Medical Center/Emory University SOM, Decatur, GA; Duke University, Durham, North Carolina; Veterans Health Administration, Case Western Reserve University School of Medicine, Cleveland, Ohio

## Abstract

**Background:**

Despite viral suppression on antiretroviral therapy (ART), people with HIV (PWH) are twice as likely to have cardiovascular disease (CVD) as those without. Here we aim to understand the association between SDH and modifiable CVD risk factors in PWH enrolled in Veterans Health Administration (VHA) healthcare, the largest US provider of HIV care with comprehensive social services.

**Table 1:**
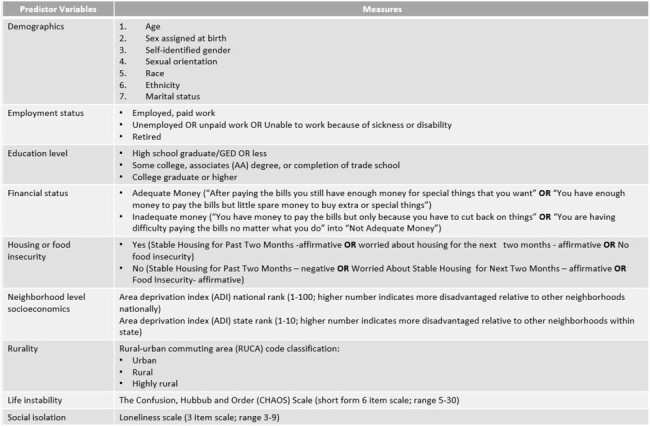
List of patient level and area level predictor variables and the associated measures used in the study.

**Methods:**

Virally suppressed PWH on ART from 4 VHA facilities completed validated questionnaires on education level, employment status, financial stability, housing and food insecurity, tobacco use, social isolation, and life instability. Demographic and laboratory data were obtained from the electronic medical record. Census block groups were used to determine area deprivation index (ADI). Baseline BP control, lipid control, and smoking status were primary outcome variables. SDH were described with medians where applicable and analyzed according to these categorical outcome measures. Comparisons were tested using Chi-square statistic and Kruskal-Wallis.

**Table 2:**
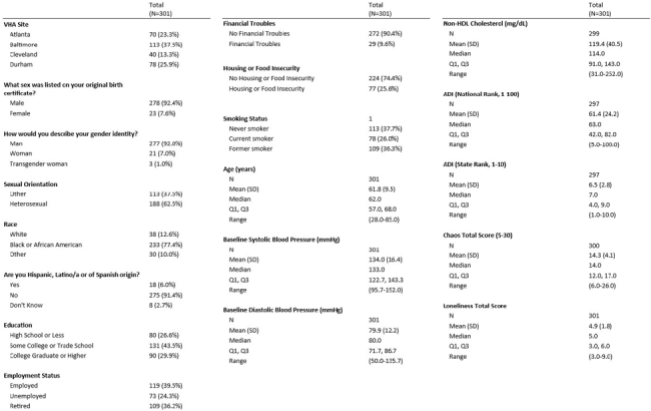
Summary of predictor variables in the overall cohort.

**Results:**

Of 301 Veterans, most were cis-gender men (92%) and identified as Black (77%), with a mean age of 62 years. Overall, 30% completed college or higher, 24% were unemployed, 10% reported no financial troubles, but 26% reported housing or food insecurity. Most were current or former smokers, 37% had uncontrolled BP, and 33% had uncontrolled lipids. Smoking status was associated with difference in educational attainment (p=0.001), life instability (p=0.03), social isolation (p=0.05), and state ADI (p=0.001), with current smokers less likely to have completed college or higher education, experiencing more instability, being more socially isolated, and living in more disadvantaged neighborhoods versus never smokers. Age differed by smoking status and lipid control (both p=0.0001), with former smokers and those with lipid control trending older. No SDH were associated with BP and lipid control.

**Figure 1:**
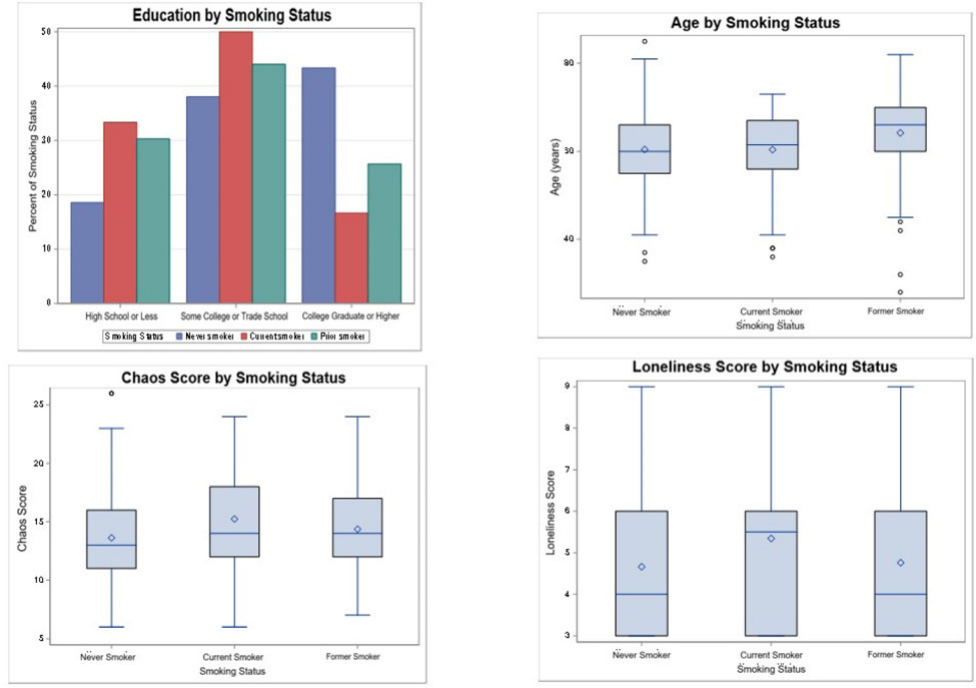
Patient-level SDH variables that correlate with smoking status.

**Conclusion:**

PWH who utilize the VHA have a high burden of CVD risk factors, but other than smoking, traditional SDH do not predict modifiable risk factors in this population. Further work is needed to understand whether VHA services mitigate the effects of factors such as race, finances, and housing insecurity that are often cited as sources of disparities.

**Figure 2:**
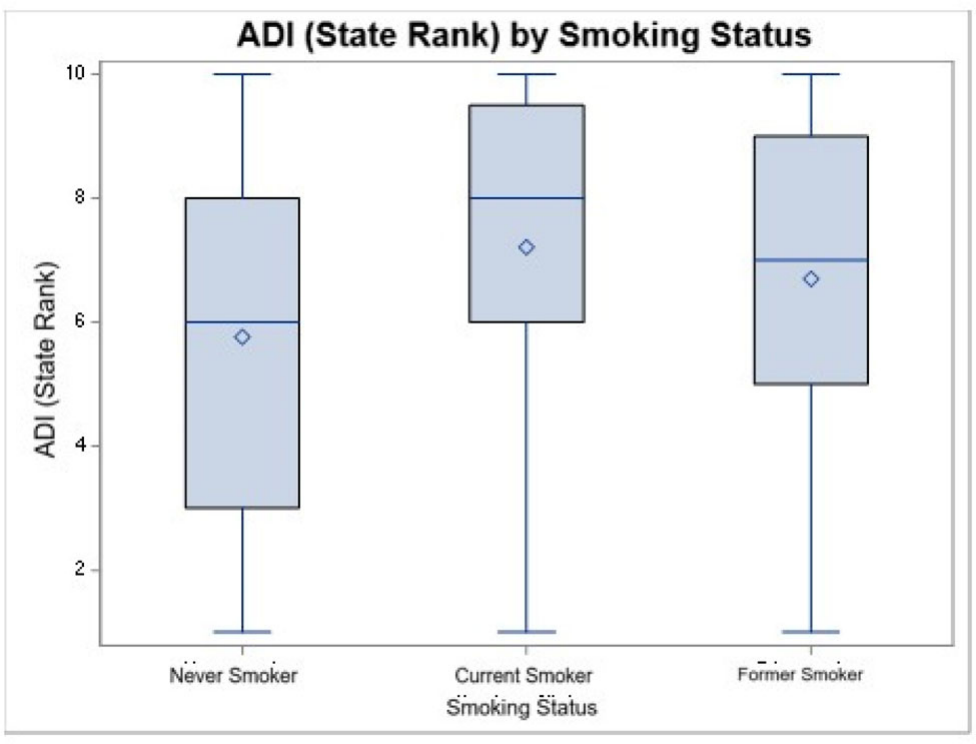
Neighborhood level SDH correlates with smoking status.

**Disclosures:**

**Hayden Bosworth, PhD**, BeBetter therapeutics: Grant/Research Support|Boehringer Ingelheim: Advisor/Consultant|Boehringer Ingelheim: Grant/Research Support|Elton John Foundation: Grant/Research Support|Esperion: Grant/Research Support|Hilton foundation: Grant/Research Support|Improved Patient Outcomes: Grant/Research Support|Merck: Grant/Research Support|NHLBI: Grant/Research Support|Novo Nordisk: Grant/Research Support|Otsuka: Grant/Research Support|Pfizer: Grant/Research Support|Preventric Diagnostics: Board Member|sanofi: Advisor/Consultant|sanofi: Grant/Research Support|Veterans Health Administration: Grant/Research Support|Walmart: Advisor/Consultant

